# Morphological signs of connective tissue dysplasia as predictors of frequent post-exercise musculoskeletal disorders

**DOI:** 10.1186/s12891-020-03698-0

**Published:** 2020-10-08

**Authors:** V. N. Nikolenko, M. V. Oganesyan, A. D. Vovkogon, Yu Cao, A. A. Churganova, M. A. Zolotareva, E. E. Achkasov, M. V. Sankova, N. A. Rizaeva, M. Y. Sinelnikov

**Affiliations:** 1 First Moscow State Medical University named after I.M.Sechenov (Sechenov University), st. Trubetskaya, 8, bld. 2, 119991 Moscow, Russia; 2 Lomonosov Moscow State University, Leninskie Gory, 1, 119991 Moscow, Russia; 3 European Osteopathic Clinical Center of the Moscow branch of the “Medical Academy of Osteopathic Education”, Gavanskaya St., 4, block 2, 199106 St. Petersburg, Russia; 4 Institute for Regenerative Medicine, Sechenov University, st. Trubetskaya, 8, bld. 2, 119991 Moscow, Russia

**Keywords:** Post-exercise musculoskeletal disorders, Connective tissue dysplasia, Morphological predictors

## Abstract

**Background:**

Connective tissue dysplasia (CTD) is a risk factor for musculoskeletal disorders. Changes caused by disorganization of collagen and elastin fibers lead to the inability of withstanding heavy mechanical stress. In clinical practice, diagnosis of these disorders depends on physical and anthropomorphic evaluation.

**Methods:**

Forty-eight patients with frequent post-exercise musculoskeletal disorders were evaluated for CTD. The control group included 36 healthy participants. Both groups were evaluated via therapeutic examination with assessment of anthropometric indicators and physical-physiological evaluation, surveying and gathering of anamnesis. Based on testing results, study participants were evaluated on CTD presence and risk factors.

**Results:**

All experimental group patients had connective tissue dysplasia of moderate and severe degree, with a total score of 49.44 ± 13.1. Certain morphological characteristics showed prevalence, allowing to determine pathognomonic predictors of high predisposition to frequent post-exercise musculoskeletal disorders. Back pain (100%), asthenic syndrome and kyphotic spinal deformation (75%), high gothic palate, hypermobility of joints and the auricles, excessive elasticity (63%), varicose veins of the lower extremities (56%) and hemorrhoids (56%), changes in the shape of the legs and temporomandibular joint (50%) showed to be significant clinical factors indicating possible connective tissue dysplasia.

**Conclusions:**

The presence of these diagnostically significant morphological signs of CTD in humans is a pathognomonic predictor of a high predisposition to frequent injuries. Their early detection helps promote proper appointment of adequate physical activity regimen and develop treatment for the underlying cause.

## Background

Physical exercise is one of the main components of a healthy lifestyle. Recently, there has been an increase in the number of musculoskeletal disorders during physical training [1] and in the number of patients with characteristic morphological signs of connective tissue dysplasia (СTD) [2–4]. CTD manifestation is due to inherited mutations of genes encoding the synthesis and spatial organization of collagen, structural proteins and protein-carbohydrate complexes, enzymes and their co-factors [2–6]. At the same time, adverse environmental factors play an important role in clinical manifestation of this condition [7, 8]. The combination of these factors leads to pathological change in elastin and collagen fibers facilitating significant restructuration of connective tissue of different severity [9], which determines the large spectrum of CTD morphological manifestations [10–15]. According to existing studies, СTD is quite common, reaching an incidence of up to 85.4% in some populations [16, 17].

CTD is characterized by a variety of clinical manifestations – from subclinical forms to polysystemic pathology [18, 19]. The classification of external and internal morphological signs of CTD has been unified [20, 21]. The combination of morphological signs is so variable that it is often difficult to integrate many individual symptoms and properly diagnose CTD [10]. Clinical interest in CTD has significantly increased largely due to the aggravating effects of this disorder on the course of almost all diseases [22]. Patients with CTD are observed by different specialists, each of whom prescribes his own treatment, which in many cases is untimely and often ineffective [23]. CTD should be considered in patients with frequent post-exercise musculoskeletal disorders, as hypersensitivity to physical exercise and predisposition to repeated injuries is a characteristic symptom of this condition [24]. It is therefore relevant to analyze the presence of morphological signs of CTD in patients with frequent post-exercise musculoskeletal disorders and injuries to determine the best course of action for prevention and treatment. In this study we assess the prevalence of morphological signs of CTD in individuals with frequent post-exercise musculoskeletal disorders in order to develop additional therapeutic-preventive measures and recommendations for choice of adequate physical activity regimen.

## Material and methods

### Participant selection

A total of 84 student and faculty volunteers form Sechenov University were included in this clinical and anthropological study, conducted in accordance to STROBE guidelines. All participants were recruited on a volunteer basis from Sechenov University, and were engaged in amateur (non-professional) running sport (track, treadmill exercises). The specter of recurrent injuries of the musculoskeletal system included ligament spraining, ruptures, joint subluxations or dislocation, which occurred during regular exercise. The subjects were split into two groups according to their state of physical health and incidence of frequent post-exercise musculoskeletal disorders. Group 1 (experimental group) included 48 subjects aged from 18 to 47 years old (average 36.38 ± 6.02 years) with frequent post-exercise musculoskeletal disorders. Group 2 (control group) was represented by 36 healthy participants, aged 18.85 ± 0.56 years, not showing any signs of post-exercise musculoskeletal disfunction.

### Clinical and anthropomorphic evaluation

All participants completed a questionnaire aimed to assess the state of connective tissue dysfunction, with analysis of all 66 morphological signs of CTD [25–27] and underwent therapeutic evaluation. Therapeutic evaluation included assessment of anthropometric indicators, such as body weight, height, arm span, chest volume, facial length and zygomatic width, length of the hand and its middle finger, the height of the lower and upper body segments and foot length. A facial index was also calculated to identify a narrow facial skeleton. Anthropomorphic proportionality was evaluated using the Verveka and Pigne indexes. The Varga and Quetelet indexes were calculated to assess body weight. Dolichostenomelia was diagnosed by the ratio of the hand and foot lengths to height, by the ratio of arm span to height, and by the ratio of the upper body segment to the lower segment. The thumb and wrist tests were performed to diagnose arachnodactyly. Hypermobility of the joints was evaluated according to Bayton’s criteria [28, 29]. Concomitant pathology of internal organs was diagnosed with ultrasound scanning, fibrogastroduodenoscopy and X-ray evaluation. Blood pressure and heart rate were documented.

### Ranking clinical significance of symptoms and CTD diagnosis

The most common symptoms were ranked according to their clinical significance based on the scale of Kadurina T.I. and Abbamukova L.N [21].. The Karudina-Abbamukova scale rates each CTD sign on a scale of 0–4 points. A total score of under 20 is characteristic of no CTD. CTD of average severity is diagnosed with a score of 21 to 40 points, and severe CTD with 41 points and more.

### Statistical analysis

The significances of differences between Group and Group 2 were determined using the independent t-test or the nonparametric Mann-Whitney U-test when variables were non-normally distributed. Anthropomorphic findings and rates of injury were compared using Pearson’s chi-squared test or Fisher’s exact test. The minimal number of subjects that needed to be enrolled in this study was calculated using power analyis. Statistical dada was calculated using RStudio software, version 1.2.1335 (RStudio, Inc., Boston, MA, USA). Results are presented as means ± standard deviation or as numbers and percentages, and statistical significance was set at *P* values < 0.05.

## Results

The minimal size sample for both groups for our dichotomous endpoint, two independent sample study (power 90%, alpha 0.05) was 14. The asthenic constitutional type was more common in Group 1. Deficit of body weight (Quetelet index ≤25) was more common in Group 1 (*p* < 0.05). Subjects in Group 1 more often had disproportionately long hands and feet in comparison with Group 2 (*p* < 0.05) (Table 1). Arachnodactyly, diagnosed by positive wrist and thumb tests was significantly more common in subjects with frequent muscular-skeletal disorders than in the control group (Table 2).

**Table 1:** The prevalence of the diagnostically significant body proportionality indexes and indexes on dolichostenomelia in individuals with frequent post-exercise musculoskeletal disorders in comparison with CG

Index	Diagnostic Values of CTD	Comparison group *n* = 36	Persons with frequent post-exercise musculoskeletal disorders *n* = 48
Quetelet Index	≤25	41,6 ± 8,2%	**62,5 ± 6,9%**^**a**^
Vargi Index	< 1,7	8,3 ± 4,5%	**31,3 ± 6,7%**^**b**^
Verveka Index	1,26-1,35	0%	**18,8** ± **5,6%**^**a**^
Pigne Index	≥30	8,3 ± 4,5%	**25,0 ± 6,3%**^**a**^
Arm swing/height	≥1,05	8,3 ± 4,5%	12,5 ± 4,8%
Upper/lower segment	<0,86	8,3 ± 4,5%	12,5 ± 4,8%
Foot length/height	> 15%	0%	**31,3 ± 6,7%**^**b**^
Wrist length/height	> 11%	0%	**25,0 ± 6,3%**^**b**^

Note. ^a^the differences are significant, ^b^the differences are highly significant

**Table 2:** The prevalence of positive tests for arachnodactyly in individuals with frequent post-exercise musculoskeletal disorders in comparison with CG

	Diagnostic Values of CTD	Comparison group *n* = 36	Persons with frequent post-exercise musculoskeletal disorders *n* = 48
Wrist test	positive	8,3 ± 4,5%	**37,5 ± 6,9%**^**a**^
Thumb test	positive	0%	**37,5 ± 6,9%**^**a**^
The length of the middle finger	> 10 см	0%	**43,8 ± 7,2%**^**a**^

Note: ^a^the differences are highly significant

Kyphotic spinal deformation with asymmetry of shoulders, shoulder blades and pelvic bones, chest deformity, flat foot combined with valgus installation, macrodactyly of the first toe significantly prevailed in Group 1 (*p* < 0.05). Back pain was present in all subjects in Group 1. The hypermobility syndrome, characterized by joint instability, “crunches” and pain during movement, repeated joint dislocations and subluxations, accompanied by ligament sprains and ruptures was common in Group 1 subjects. Subjects in Group 1 had a narrow facial skeleton, malocclusion and a high gothic palate (Table 3***)***.

**Table 3:** Osteo-articular signs in individuals with frequent post-exercise musculoskeletal disorders in comparison with CG

Osteo-articular signs of CTD	Comparison group *n* = 36	Persons with frequent post-exercise musculoskeletal disorders *n* = 48
Scoliosis	41,6 ± 8,2%	43,8 ± 7,2%
Kyphosis	8,3 ± 4,5%	**75,0** ± 6,3%**
Kyphoscoliosis	0%	25,0 ± 6,3%**
«Flat» back	0%	6,3 ± 3,5%
Shoulder blades’ asymmetry	0%	50,0 ± 7,2%**
Shoulder asymmetry	25,0 ± 7,2%	87,5 ± 4,8%**
Pelvic bones’ asymmetry	0%	50,0 ± 7,2%**
Pterygoid shoulder blades	8,3 ± 4,5%	12,5 ± 4,8%
Infundibular chest deformity	0%	**12,5 ± 4,8%**^**a**^
Keel-shaped chest deformity	0%	**12,5 ± 4,8%**^**a**^
Flatfoot	50,0 ± 8,3%	43,8 ± 7,2%
Feet valgus installation	8,3 ± 4,5%	43,8 ± 7,2%**
X- and O-shaped legs	0%	50,0 ± 7,2%**
Macrodactyly of the first toe	0%	31,3 ± 6,7%**
Pain in the spine	25,0 ± 7,2%	100%**
Joint hypermobility	0%	62,5 ± 6,9%**
“Crunch” in the joints	25,0 ± 7,2%	62,5 ± 6,9%**
“Crunch” in the temporomandibular joint	16,6 ± 6,2%	50,0 ± 7,2%**
Arthralgia	0%	50,0 ± 7,2%**
Joint dislocations and subluxations	0%	**12,5 ± 4,8%**^**a**^
Ligament sprains and ruptures	25,0 ± 7,2%	100%**
Bone fractures	8,3 ± 4,5%	50,0 ± 7,2%**
Narrow facial skeleton	0%	**12,5 ± 4,8%**^**a**^
Wide-set eyes	0%	6,3 ± 3,5%
Gothic high palate	0%	62,5 ± 6,9%**
Malocclusion	0%	**18,8** ± **5,6%**^**a**^

^a^statistically significant differences

**the differences are highly significant

Keloid scars and hemorrhagic syndrome (petechiae), hyperpigmentation along the spinous processes, atrophic skin striae, and telangiectasia were more common Group 1 (*p* < 0.05). Most in Group 1 had increased brittleness, softness in nails as well as disorders of the homogeneous structure of the nail plate (*p* < 0.05). Thin, brittle hair, areas of alopecia, auricles with excessive elasticity were common in Group 1 (*p* < 0.05). Patients in Group 1 showed significantly higher rates of muscular pathology (hypotrophy, muscle hypotension, diastasis of the rectus abdominis muscles and abdominal hernias) (*p* < 0.05) (Table 4***).***

**Table 4:** Ectodermal signs in individuals with frequent post-exercise musculoskeletal disorders in comparison with CG

Signs of CTD	Comparison group *n* = 36	Persons with frequent post-exercise musculoskeletal disorders *n* = 48
Thin skin	8,3 ± 4,5%	**25,0 ± 6,3%**^**a**^
Nosebleeds	8,3 ± 4,5%	12,5 ± 4,8%
Keloid scars	0%	**25,0 ± 6,3%**^**a**^
Petechia	0%	**18,8** ± **5,6%**^**a**^
Skin hyperpigmentation above the spine	0%	**12,5 ± 4,8%**^**a**^
Atrophic striae	8,3 ± 4,5%	37,5 ± 6,9%**
Teleangiectasia	0%	**12,5 ± 4,8%**^**a**^
Nails (soft/fragile/exfoliated)	33,3 ± 7,9%	87,5 ± 4,8%**
Hair (thin/brittle/areas of alopecia)	8,3 ± 4,5%	81,3 ± 5,6%**
Auricles (soft/rolled into a tube)	8,3 ± 4,5%	62,5 ± 6,9%**
Myotonic syndrome	8,3 ± 4,5%	18,8 ± 5,6%
Diastasis of rectus abdominis muscle	0%	**18,8** ± **5,6%**^**a**^
Hernia	0%	**12,5 ± 4,8%**^**a**^

^a^statistically significant differences

**the differences are highly significant

Cardiovascular pathology (mitral valve prolapse, vascular dystonia, rate of tachycardia incidents, lower overall systolic pressure, varicose veins, hemorrhoids), ophthalmic manifestations (medium severity myopia) and gastrointestinal pathologies (biliary dyskinesia, failure of the stomach cardia and gastroesophageal reflux syndrome) were more common in Group 1 (Table 5). Asthenic syndrome (decreased working capacity, increased fatigue, sleepiness, general weakness, especially in the mornings) and pain were often observed in the patients with frequent post-exercise musculoskeletal disorders (*p* < 0.05).

**Table 5:** Internal signs in individuals with frequent post-exercise musculoskeletal disorders in comparison with CG

Signs of CTD	Comparison group *n* = 36	Persons with frequent post-exercise musculoskeletal disorders *n* = 48
Mitral valve prolapse	8,3 ± 4,5%	**25,0 ± 6,3%**^**a**^
Vascular dystonia	41,6 ± 8,2%	**75,0** ± **6,3%**^**a**^
Pulse, beats/min	67,13 ± 5,62	**78,63 ± 6,53**^**a**^
Systolic blood pressure	117,9 ± 4,51	**108,3 ± 6,12**^**a**^
Diastolic blood pressure	72,66 ± 6,71	69,3 ± 4,58
Lower extremities’ varicose veins	0%	**56,3 ± 7,2%**^**b**^
Hemorrhoids	8,3 ± 4,5%	**56,3 ± 7,2%**^**b**^
Average myopia	58,3 ± 8,2%	**75,0** ± **6,3%**^**a**^
Astigmatism	16,6 ± 6,2%	12,5 ± 4,8%
Biliary dyskinesia	16,6 ± 6,2%	**62,5 ± 6,9%**^**b**^
Gastroesophageal reflux	8,3 ± 4,5%	**68,8 ± 6,7%**^**b**^
Chronic esophagitis	0%	**68,8 ± 6,7%**^**b**^
Asthenic syndrome	8,3 ± 4,5%	**75,0** ± **6,3%**^**b**^

Note. ^a^the differences are significant, ^b^the differences are highly significant

The most common and diagnostically significant symptoms according to the Kadurina-Abbamukova scale [21] were kyphotic spinal deformation, myopia, asthenic syndrome and back pain. The total score of each individual patient in Group 2 was less than 20 points, which was not characteristic of CTD. Patients in Group 1 were diagnosed with medium severity CTD in 37.5% and severe CTD in 62.5% of cases. The total score of CTD signs in these patients was significantly higher (49,44 ± 13,1) compared to the control group (11,33 ± 3,29) (Table 6).

**Table 6:** Ranking of the most common morphological signs depending on their clinical significance in individuals with frequent post-exercise musculoskeletal disorders based on the scale of Kadurina T.I. and Abbamukova L.N

Signs of CTD	Prevalence, %	Clinical significance grade (Kadurina & Abbamukova Scale)
Quetelet Index≤25	62,5 ± 6,9%	2
**Kyphotic spinal deformation**	**75,0** ± **6,3%**	**4**
Shoulder asymmetry	87,5 ± 4,8%	2
Shoulder blade asymmetry	50,0 ± 7,2%	2
Asymmetry of the pelvic bones	50,0 ± 7,2%	2
**X- and O-shaped legs**	**50,0 ± 7,2%**	**3**
**Pain in the spine**	**100%**	**4**
**Joint hypermobility moderate**	**62,5 ± 6,9%**	**3**
**Gothic high palate**	**62,5 ± 6,9%**	**3**
«Crunch» in the joints	62,5 ± 6,9%	2
**“Crunch” in the temporomandibular joint**	**50,0 ± 7,2%**	**4**
Nails (soft/ brittle/exfoliated)	87,5 ± 4,8%	2
Hair (thin/brittle/areas of alopecia)	81,3 ± 5,6%	2
**Auricles (soft/rolled into a tube)**	**62,5 ± 6,9%**	**3**
Vascular dystonia	75,0 ± 6,3%	2
**Varicose veins of the lower extremities**	**56,3 ± 7,2%**	**3**
**Hemorrhoids**	**56,3 ± 7,2%**	**3**
**Myopia of an average degree**	**75,0** ± **6,3%**	**3**
Biliary dyskinesia	62,5 ± 6,9%	2
Gastroesophageal reflux	68,8 ± 6,7%	2
Chronic esophagitis	68,8 ± 6,7%	2
**Asthenic syndrome**	**75,0** ± **6,3%**	**4**

## Discussion

All subjects with frequent post-exercise musculoskeletal disorders in this study were diagnosed with CTD. This underlines the overlooked significance of CTD in clinical medicine [30]. Such patients should observe a specific physical regimen with proper alternation of exercise and rest [11, 23]. The necessity of adequate physical activity and training pace has long been established [31]. Patients with CTD are recommended to perform regular aerobic exercises 3 times a week for 40–60 min (swimming, walking, moderate running, cycling, badminton, bowling, table tennis). Patients with CTD have been advised no to partake in activities such as ballet, group sports associated with a high risk of injury and weight-lifting [18, 23, 32].

Our study was aimed at assessing the clinical and morphological signs which can suggest the presence of CTD in otherwise undiagnosed patients. Our results have shown that the asthenic constitutional type was a common morphological feature in patients with CTD. This phenomenon has been recognized by several population-based studies [30, 33, 34]. It is important to further consider the phenotypic manifestations of CTD in the form of appropriate clinical syndromes. Most often this pathology is characterized by osteoarticular changes (Table 3) [3, 4].

One of the important morphological signs of CTD, characterizing the involvement of the skeletal system, is dolichostenomelia (lengthening of the extremities), which is determined by measuring the tubular bones and estimating indexes [35]. Our study has shown that up to 63% of patients with frequent post-exercise musculoskeletal disorders present with a Quetelet index (BMI) ≤25, 31% with Vargi Index < 1.7, 25% with Pigne Index ≥30 and 19% with Verveka Index between 1.26 and 1.35. Limb changes in CTD often present with arachnodactyly (long, thin, “spider” fingers) [36], which, according to our study along with muscular and vascular symptoms was significantly more common in patients with frequent muscular-skeletal disorders. These findings hint towards presence undifferentiated connective tissue disease (UCTD) in the experimental group.

Ectodermal manifestations (Table 4) in examined patients were characterized by a more frequent presence of thin skin with a well-visible network of subcutaneous vasculature located on the chest, back and limbs. In a significant percentage of cases, manifestations of the vascular syndrome were seen. This is an important aspect, due to the presence of vasculature pathology in many severe connective tissue diseases, which may help early detection of underlying pathology [37–39].

Consistent with existing data, many patients with frequent post-exercise musculoskeletal disorders have shown to have specific gastrointestinal symptoms. Therefore, gastrointestinal tract pathology was significantly more often detected in the experimental group in the form of biliary dyskinesia, failure of stomach cardia and gastroesophageal reflux syndrome (Table 5). This is characteristic of many underlying connective tissue diseases, such as eosinophilic disorders [40], gastrointestinal vasculitis [41], Ehlers-Danlos syndromes [42], and others [43, 44].

The presence of the discussed diagnostically significant morphological signs of CTD in humans is a pathognomonic predictor of high susceptibility to frequent post-exercise musculoskeletal disorders and requires compliance with additional therapeutic and preventive recommendations. Our study showed that surveying and anthropomorphic evaluation has allowed to determine predictors of high predisposition to frequent post-exercise musculoskeletal disorders. It is important to properly address these symptoms and anthropomorphic presentations in order to build a personalized approach to physical exercise regimen and lifestyle changes in this group of patients. Improper physical training in patients with CTD can lead to significant health problems. More so, early screening for CTD can help determine a large specter of predisposed diseases.

Osteopathy is recommended as part of CTD treatment. This method, restoring impaired structural-anatomical relationships between various organs and parts of the body, contributes to the normalization of body functions [45]. Regularly conducted courses of osteopathy improve reflex activity and metabolic processes, leading to strengthening of connective tissue [46]. Further research should expand upon these findings, forming a standardized approach to early diagnosis of CTD in adolescents.

## Conclusion

All patients with frequent post-exercise musculoskeletal disorders showed signs of CTD. The analysis of the morphological prevalence depending on clinical significance allowed us to determine pathognomonic predictors of high predisposition to injuries. Their early detection can promote the timely appointment of adequate physical activity regimen and therapy of the underlying cause. Based on our research results, we recommend proper evaluation of patients with frequent post-exercise musculoskeletal disorders, as this may be the only symptom of an underlying connective tissue disorder.

## Supplementary information

**Additional file 1.**

**Additional file 2.**

## Data Availability

The datasets used during the current study are available from the corresponding author on reasonable request.

## Abbreviation

CTD: Connective tissue dysplasia
