# Supplementary material for: Morphological signs of connective tissue dysplasia as predictors of frequent post-exercise musculoskeletal disorders
Source: BMC Musculoskelet Disord. 2020 Oct 8;21:660. doi: 10.1186/s12891-020-03698-0 (PMC7545870; doi:10.1186/s12891-020-03698-0)
Supplement: Supplementary file 2 — Additional file 2. [file 12891_2020_3698_MOESM2_ESM.pdf]

QUESTIONNAIRE №

I. PATIENT FORM

Full name

Mobile phone **Sex** **F** **M** **Age**

1. **Please answer the following questions truthfully. Be as thorough as possible:**
   1) **Do you have any active complaints about your wellbeing?**
   2) **How would you describe the presence of pain in your life.**
   3) **How would you best describe your health?**
   4) **Pease thoroughly describe your physical exercise regimen.**

2. **Please answer the following questions by using a scale from 1-10, where 1 is "Strongly disagree" and 10 is "Strongly agree":**
   1) **I experience pain in my spine.**
   2) **My joint "crunch".**
   3) **I have had ligament sprains and/or ruptures.**
   4) **I have experienced bone injury.**
   5) **I have experienced bone fractures.**
   6) **I feel a "crunching" and/or "grinding" sensation when I chew.**
   7) **I feel pain in my joints.**
   8) **I have had dislocated joints.**
   9) **I experienced joint subluxations.**
   10) **I am often tired.**
   11) **I do not get enough sleep.**
   12) **I wake up tired.**

Please rate (1-10):

1) **QUALITY OF LIFE**	1	2	3	4	5	6	7	8	9	10
2) **OVERALL HEALTH**	1	2	3	4	5	6	7	8	9	10

II. EXAMINATOR FORM

1. **Primary assesement**:

**Spine pain**		**"Crunch" in the temporomandibular joint**	
**"Crunch" in the joints**		**Arthralgia**	
**Ligament sprains and ruptures**		**Joint dislocations and subluxations**	
**Bone fractures**		**Asthenic syndrome**	
**Blood pressure**		**Heart rate**	

2. **Anamnesis:**

3. **The type of exercise that caused the injury:**

4. **Anthropometric measurements:**

**Weight**		**Height**		**Chest volume**	
**Quetelet Index**		**Varga Index**		**Verveque Index**	
**Pigne Index**		**Arm swing**		**Arm swing/height**	
**Foot height**		**Foot length**		**Foot length/height**	
**Face height**		**Zygomatic width**		**Facial index**	
**Wrist length**		**Middle finger length**		**Wrist length/height**	

5. **Osteo-articular signs:**

**Scoliosis**		**Flatfoot**	
**Kyphosis**		**Feet valgus installation**	
**Kyphoscoliosis**		**X- and O-shaped legs**	
**«Flat» back**		**Macrodactyly of the first toe**	
**Shoulder blades' asymmetry**		**Joint hypermobility (Bayton's criteria)**	
**Shoulder asymmetry**		**Wide-set eyes**	
**Pelvic bones' asymmetry**		**Gothic high palate**	
**Pterygoid shoulder blades**		**Malocclusion**	
**Infundibular chest deformity**		**Keel-shaped chest deformity**	
**Micrognathia**		**Nasal septum curvature**	
**Hammer toes**		**Hip displasia**	
**Wrist test**		**Thumb test**	

6. **Myopathic and ectodermal signs:**

**Myotonic syndrome**		**Skin hyperpigmentation above the spine**	
**Rectus muscles diastasis**		**Atrophic striae**	
**Recurrent hernia**		**Teleangiectasia**	
**Thin hyperelastic skin**		**Nails (soft/fragile/exfoliated)**	
**Nosebleeds**		**Hair (thin/brittle/areas of alopecia)**	
**Keloid scars**		**Auricles (soft/rolled into a tube)**	
**Petechia**		**Ecchymosis**	
**Bluish / blue sclera**		**The "tissue paper" symptom**	

7. <u>Internal signs:</u>

**Mitral valve prolapse**		**Astigmatism**	
**Vascular dystonia**		**Cataract**	
**Pulse, beats/min**		**Glaucoma**	
**Systolic blood pressure**		**Lens subluxation**	
**Diastolic blood pressure**		**Strabismus**	
**Lower extremities' varicose veins**		**Myopia**	
**Hemorrhoids**		**Biliary dyskinesia**	
**Esophagus Diverticula**		**Chronic esophagitis**	
**Hiatal hernia**		**Gastroesophageal reflux**	
**Kidney, liver cysts**		**Organ ptosis**	
**Urinary incontinence**		**Lungs Emphysema**	

III.STUDY NOTES.

(include date of note)
